# True and False DRM Memories: Differences Detected with an Implicit Task

**DOI:** 10.3389/fpsyg.2012.00310

**Published:** 2012-08-27

**Authors:** Maddalena Marini, Sara Agosta, Giuliana Mazzoni, Gianfranco Dalla Barba, Giuseppe Sartori

**Affiliations:** ^1^University of Modena and Reggio EmiliaReggio Emilia, Italy; ^2^Italian Institute of TechnologyRovereto, Italy; ^3^University of HullHull, UK; ^4^University of TriesteTrieste, Italy; ^5^Dipartimento di Psicologia Generale, University of PaduaPadua, Italy

**Keywords:** autobiographical IAT, false memories, DRM paradigm, implicit associations, implicit level

## Abstract

Memory is prone to illusions. When people are presented with lists of words associated with a non-presented critical lure, they produce a high level of false recognitions (false memories) for non-presented related stimuli indistinguishable, at the explicit level, from presented words (DRM paradigm). We assessed whether true and false DRM memories can be distinguished at the implicit level by using the autobiographical IAT (aIAT), a novel method based on indirect measures that permits to detect true autobiographical events encoded in the respondent’s mind/brain. In our experiment, after a DRM task participants performed two aIATs: the first aimed at testing implicit memory for presented words (true-memories aIAT) and the second aimed at evaluating implicit memory for critical lures (false-memories aIAT). Specifically, the two aIATs assessed the association of presented words and critical lures with the logical dimension “true.” Results showed that the aIAT detected a greater association of presented words than critical lures with the logical dimension “true.” This result indicates that although true and false DRM memories are indistinguishable at the explicit level a different association of the true and false DRM memories with the logical dimension “true” can be detected at the implicit level, and suggests that the aIAT may be a sensitive instrument to detect differences between true and false DRM memories.

## Introduction

It is known that human memory is prone to various kinds of distortions and illusions (Roediger, 1996; Schacter, 1999; Loftus, 2003). Among these memory errors, many researchers have focused on false recognition, whereby people incorrectly claim that they have recently seen or heard a stimulus they have not encountered (Underwood, 1965). It has been shown that, in contrast to deception, memory illusions are often unaccompanied by a subjective feeling that people are responding untruthfully. On the contrary, memory illusions such as those produced in the Deese–Roediger–McDermott paradigm (DRM, Deese, 1959; Roediger and McDermott, 1995), are accompanied by a sense of recollection which, at the explicit level, makes them indistinguishable from true memories. DRM false memories are obtained by presenting lists of words related to a non-presented critical lure. The probability of recalling or recognizing the critical lure is usually quite high (Roediger and McDermott, 1995; Balota et al., 1999; Stadler et al., 1999; Budson et al., 2002). Previous findings have shown that critical lures seem to elicit the same recollective quality (*remember* judgments) of presented items (e.g., Roediger and McDermott, 1995) and participants are even able to state in which voice they heard the non-presented critical lure when half list items had been presented by a female voice and half by a male voice (Payne et al., 1996).

Imaging evidence, however, has revealed some notable differences between true and false memories. In terms of overall differences, true memories have been strongly associated with the medial temporal lobe (MTL) regions (e.g., Cabeza et al., 2001; Okado and Stark, 2003; Slotnick and Schacter, 2004; Dennis et al., 2012), whereas false memories have been associated with the pre-frontal cortex (PFC) and parietal regions (e.g., Schacter et al., 1996, 1997; Cabeza et al., 2001; Slotnick and Schacter, 2004; Garoff-Eaton et al., 2007). For example, Cabeza et al. (2001), using fMRI in combination with a verbal variant of the DRM paradigm, have shown that true recognition was associated with a higher activation of the parahippocampal gyrus (PHG), whereas false recognition was associated with a higher activation of the orbito-PFC. These results have been corroborated by Okado and Stark (2003). It has been proposed that MTL activity related to true-memories reflects mainly a recollection mechanism, whereas frontoparietal activity related to false memories reflects mainly a familiarity mechanism (Kim and Cabeza, 2007). In agreement with this view, patient studies and neuroimaging studies showed that MTL regions such as the hippocampus and posterior PHG are critical for recollection (for a review, see Yonelinas, 2002; Eichenbaum et al., 2007), whereas frontoparietal regions such as the anterior (aPFC) and dorsolateral PFC (DLPFC) are critical for familiarity (Cansino et al., 2002; Yonelinas et al., 2005; Duarte et al., 2010). Similarly, Moritz et al. (2006) using the DRM has shown that an increase in confidence at recognition was associated with an activation in the cingulate cortex along with medial temporal regions, whereas increments in doubt (i.e., when people showed low-confident judgments) were related to activation in the superior posterior parietal cortex. Taken together, these results show that, although at an explicit level true and false recognitions may be indistinguishable, they are processed by different neural substrates. Thus, at some level neurological markers can differentiate between true and false recognitions, suggesting that true and false DRM memories might also be distinguished at the implicit level, when individuals are not directly asked to recognize true/false items.

A novel method based on indirect measures that might distinguish between true and false DRM memories is the autobiographical IAT (aIAT; Sartori et al., 2008). The aIAT is a variant of the Implicit Association Test (IAT; Greenwald et al., 1998) which investigates the memory representation of autobiographical events and is specifically aimed at accurately detecting autobiographical events encoded in the respondent’s mind/brain.

The aIAT includes stimuli belonging to four categories. Two categories are logical categories and are represented by statements which are always true (e.g., *I am in front of a computer)* or always false (e.g., *I am in front of a television*) for the respondent. Two other categories are represented by alternative versions of an autobiographical event (e.g., *I went to Paris for Christmas* vs. *I went to London for Christmas*), only one of the two being true. The true autobiographical event can be identified because it gives rise to faster RTs when it shares the same motor response as true statements.

The accuracy of the aIAT in detecting the true autobiographical event is very high, both at the group level and at the individual level (about 91%), and it is a flexible tool because it can evaluate any type of autobiographical memory that may be described with a sentence. Moreover, in comparison with other lie-detection or memory-detection techniques (e.g., fMRI) it has several more advantages: it can be administered quickly (10–15 min), it is based on an unmanned analysis (no training for the user is necessary), it requires low-tech equipment (a standard PC is sufficient), and it can also be administered remotely to many participants (e.g., via the web) for screening purposes.

The main objective of the present study was to determine whether differences between true and false DRM memories can be detected with the aIAT (Sartori et al., 2008). To this end, we used eight word lists developed by Deese (1959) and modified by Roediger and McDermott (1995) in order to produce false recognitions of non-presented critical lures with high levels of probability. The DRM procedure consisted of an encoding stage, in which participants heard semantically associated word lists (e.g., butter, food, eat, sandwich, rye, etc.), each followed by a recognition task which included previously presented words (e.g., sandwich), the non-presented critical lure, which is the strongest word associated with the presented items (e.g., bread), and unrelated non-presented distracters (e.g., dog). In accordance with several studies (e.g., Roediger and McDermott, 1995), we expected to find high false-alarm rates for the non-presented critical lures, whereas false-alarm rates for the non-presented distracters would be low. In the DRM recognition tasks participants were asked to give Present/Absent responses (i.e., they had to classify words as Present when the item had been presented and as Absent when the item had not been presented during the encoding stage).

The DRM task was followed by the two aIAT tasks. One aIAT evaluated the association of the presented items with the logical dimension “true” (true-memories aIAT), whereas the second aIAT evaluated the association of the critical lures with the logical dimension “true” (false-memories aIAT). By comparing the results of the two aIATs one could observe if true memories (presented items in the true-memories aIAT) and false memories (critical lures in the false-memories aIAT) were encoded differently as suggested by the results of neuroimaging studies. Therefore, if differences between presented items and critical lures exist at the implicit level and can be identified by the aIAT, the two main indexes in this instrument should be different for the true-memories aIAT and the false-memories aIAT. Specifically, both the *aIAT effect* (i.e., the difference in average RTs between the congruent and the incongruent condition) and the *D index* (Greenwald et al., 2003); i.e., the difference between the incongruent and congruent conditions within each aIAT measuring accuracy at the level of the individual) would reveal a difference between true and false DRM memories. On the contrary, if the aIAT is based on the individual’s awareness that the critical lure was presented or if presented items and critical lures are characterized by a non-distinguishable level of activation at the implicit cognitive level, then the aIAT would be ineffective in detecting any difference between presented items and critical lures.

## Materials and Methods

### Participants

Thirty-six undergraduate students (aged 19–33, mean = 23.94, SD = 2.97; 18 females) volunteered to take part in the experiment. All participants were informed about the general nature of the research and voluntary option to participate. Only participants that gave their informed consent participated to the experiment. The study was approved by the Ethics Committee of the Department of General Psychology of the University of Padua.

### Experimental paradigm, procedure, and stimuli

The experimental paradigm and the structure of the tasks are schematically shown in Table 1. All participants were tested in two sessions. In the first session, they performed the DRM task and in the second session they performed two aIATs.

**Table 1:** **Scheme of the experimental paradigm and structure of the tasks**.

First session	Second session	
DRM task	True-memories aIAT	False-memories aIAT
**Stage 1: encoding task** eight lists of 15 words	**Block 1: logical discrimination** 10 true/false factual statements (20 trials)	**Block 1: logical discrimination** 10 true/false factual statements (20 trials)
	**“A” key: true category**	**“A” key: true category**
	(true factual sentences; e.g., I am in front of a computer)	(true factual sentences; e.g., I am in front of a computer)
	**“L” key: false category**	**“L” key: false category**
	(false factual sentences, e.g., I am in front of a television)	(false factual sentences, e.g., I am in front of a television)
**Stage 2: recognition test**	**Block 2: initial episodic discrimination**	**Block 2: initial episodic discrimination**
eight lists of eight words (three targets, one critical lure and four distracters)	10 sentences relating to the DRM paradigm (20 trials)	10 sentences relating to the DRM paradigm (20 trials)
**“N” key: presented words**	**“A” key: present category**	**“A” key: present category**
(targets; e.g., BUTTER)	(sentences referred to presented and recognized *targets*; e.g., I heard the word BUTTER)	(sentences referred to non-presented *critical lures* recognized as presented; e.g., I heard the word BREAD)
**“M” key: non-presented words**	**“L” key: absent category**	**“L” key: absent category**
(critical lures and distracters; e.g., BREAD and TIRED)	(sentences related to non-presented *distracters*; e.g., I heard the word TIRED)	(sentences related to non-presented *distracters*; e.g., I heard the word TIRED)
	**Block 3: double categorization or congruent condition**	**Block 3: double categorization or congruent condition**
	10 true/false factual statements and 10 sentences relating to the DRM paradigm (60 trials)	10 true/false factual statements and 10 sentences relating to the DRM paradigm (60 trials)
	**“A” key: true/present category**	**“A” key: true/present category**
	(both true factual statements and sentences referred to presented and recognized *targets*; e.g., I am in front of a computer and I heard the word BUTTER)	(both true factual statements and sentences referred to non-presented *critical lures* recognized as presented; e.g., I am in front of a computer and I heard the word BREAD)
	**“L” key: false/absent category**	**“L” key: false/absent category**
	(both false factual statements and sentences related to non-presented *distracters*; e.g., I am in front of a television and I heard the word TIRED)	(both false factual statements and sentences related to non-presented *distracters*; e.g., I am in front of a television and I heard the word TIRED)
	**Block 4: reversed episodic discrimination**	**Block 4: reversed episodic discrimination**
	10 sentences relating to the DRM paradigm (40 trials)	10 sentences relating to hearing to the DRM paradigm (40 trials)
	**“A” key: absent category**	**“A” key: absent category**
	(sentences related to non-presented *distracters*; e.g., I heard the word TIRED)	(sentences related to non-presented *distracters*; e.g., I heard the word TIRED)
	**“L” key: present category**	**“L” key: present category**
	(sentences referred to presented and recognized *targets*; e.g., I heard the word BUTTER)	(sentences referred to non-presented *critical lures* recognized as presented; e.g., I heard the word BREAD)
	**Block 5: reversed double categorization or incongruent condition**	**Block 5: reversed double categorization or incongruent condition**
	10 true/false factual statements and 10 sentences relating to the DRM paradigm (60 trials)	10 true/false factual statements and 10 sentences relating to the DRM paradigm (60 trials)
	**“A” key: true/absent category**	**“A” key: true/absent category**
	(both true factual statements and sentences related to non-presented *distracters*; e.g., I am in front of a computer and I heard the word TIRED)	(both true factual statements and sentences related to non-presented *distracters*; e.g., I am in front of a computer and I heard the word TIRED)
	**“L” key: false/present category**	**“L” key: false/present category**
	(both false factual statements and sentences referred to presented and recognized *targets*; e.g., I am in front of a television and I heard the word BUTTER)	(both false factual statements and sentences referred to non-presented *critical lures* recognized as presented; e.g., I am in front of a television and I heard the word BREAD)

The DRM task consisted of two stages. In the first stage eight lists of 15 Italian words were auditorily presented at a rate of one word every 2 s (see Ciaramelli et al., 2006). These lists were the Italian translation and adaptation of the lists of semantic associates drawn up by (Stadler et al., 1999). Each list converged on a critical lure representing the gist of the list (e.g., the word *bread* for a list comprising *butter, food, eat, sandwich, rye*, etc.). Immediately after the presentation of each list, participants were administered a recognition test with eight words, which included three words presented in the previous list (targets), and five non-presented words. Targets were the items in serial position 1, 8, and 10 from that list. The non-presented words included the critical lure of the presented list (critical lure) and unrelated distracters, that is, the critical lure of a non-presented list (control lure) and the items in serial position 1, 8, 10 of the same non-presented list (control target). Considering all eight word lists, the total number of items used for the eight recognition tests included 64 words, 24 presented, and 40 non-presented. The order of presentation of the eight words in each recognition test was randomized. In the DRM recognition test, participants were instructed to respond by pressing one of two keys of a keyboard: the Presented key (N key) if the word was presented and the Absent key (M key) if the word was not presented.

After 5 min, participants performed the aIATs. The first aIAT (true-memory aIAT) evaluated the association of the targets (true memories) with the logical dimension “true,” whereas the second aIAT (false-memories aIAT) assessed the association of the critical lures (false memories) with the logical dimension “true.”

Participants were asked to categorize 10 factual statements (which are the same across all aIATs) belong to logical dimensions “true” or “false” and 10 statements referring to the DRM lists (which changed participant by participant according to the result of the recognition memory phase). Each aIAT consisted of five separate blocks of categorization trials. In each trial, a statement was presented on the center of a computer monitor. Participants were requested to categorize the statements as quickly and accurately as possible by pressing one of two labeled keys.

In Block 1 (20 trials; logical discrimination), participants classified factual statements as true or false. Factual statements referred to the activities of the participants during the experiment. They were asked to press the A key (True category) if the factual sentence was true for them (e.g., I am in front of a computer) and the L key (False category) if the factual sentence was false for them (e.g., I am in front of a television).

In Block 2 (20 trials; initial episodic discrimination), participants categorized statements relative to words that they could or could not have heard during the presentation stage of the DRM as present or absent. They pressed the A button (Present category) to classify the sentences related to words that they had heard and L button (Absent category) to classify the sentences referred to words that they had not heard.

In Block 3 (60 trials; double categorization or *congruent* condition), participants categorized both factual statements and statements referring to words of the DRM lists. They were asked to press the A key (True/Present category) to classify both the true factual statements and sentences related to words that they had heard in the prior DRM task, whereas the L key (False/Absent category) was used to classify both false factual statements and sentences related to words that they had not heard in the DRM paradigm.

In Block 4 (40 trials; reversed episodic discrimination), participants classified only statements referring to words of the DRM lists as in Block 2 but using a different key configuration. They were asked to press the A key for the sentences related to word that they had not heard in the DRM paradigm (Absent category) and the L key for sentences related to words that they had heard in the prior DRM paradigm (Present category).

In Block 5 (60 trials; reversed double categorization or *incongruent* condition), participants classified both factual statements and statements referring to words of the DRM lists as in Block 3, but in this condition they pressed the A key (True/Absent category) for true factual statements and the sentences related to word that they had not heard in the DRM paradigm, the L key (False/Present category) for false factual statements and sentences related to words that they had heard in the prior DRM paradigm.

For the *true-memories aIAT*, we defined as Present category the statements referring to presented and correctly recognized targets in the DRM task (e.g., I heard the word *BUTTER*), whereas in the in the *false-memories aIAT* we arbitrarily defined as Present category the sentences referring to the non-presented critical lures and incorrectly recognized as presented in the DRM paradigm (e.g., I heard the word *BREAD*). In both aIATs, we defined as Absent category the statements referring to unrelated distracters correctly recognized in the DRM recognition test as non-presented (e.g., I heard the word *TIRED*).

Notably, in both aIATs the Absent category indicated false events (i.e., sentences referring to having heard non-presented and unrelated distracters), whereas the Present category indicated true events in the true-memories aIAT (i.e., sentences referring to hearing to presented targets) and false events in the false-memories aIAT (i.e., sentences referring to hearing to non-presented critical lures).

Reminder labels in the form of category names remained on the monitor for the entire duration of each block. Factual true-false statements and sentences related to present-absent words in the DRM paradigm were alternated in Blocks 3 and 5.

In order to counterbalance the presentation order in the aIAT, half of the participants performed first the *false-memories aIAT* task, followed by the *true-memories aIAT*, whereas for the other half the order was reversed.

## Results

### Recognition

Proportions of recognition were submitted to a repeated-measures analysis of variance (ANOVA) with type of recognition (target hit rates, false-alarm rates to critical lures, false-alarm rates to unrelated distracters – these latter collapsed across control targets, and control lures) as the within-subject factor.

A main effect of type of recognition was obtained: *F*(2,70) = 852.849, *p *< 0.001, η^2^ = 0.961. Bonferroni-corrected pairwise comparisons showed a significant difference between hit rates for target items and false-alarm rates for unrelated distracters [0.90 vs. 0.01%; MD = 0.888, *p *< 0.001, 95% CI = (0.847, 0.928)], as well as between false-alarm rates for critical lures and false-alarm rates for unrelated distracters [0.84 vs. 0.01%; MD = 0.830, *p *< 0.001, 95% CI = (0.770, 0.890)]. The test did not reveal a significant difference between recognition rates for target and critical lures (90 vs. 84%; *p *= 0.192).

This pattern of results indicated that recognition in the DRM task was high and not significantly different for both presented targets and non-presented critical lures. False alarms for non-presented distracters were significantly lower than for critical lures. Therefore, at an explicit level false memories for the critical lures were considered similar to true memories for the presented targets.

### Autobiographical IAT

Reaction times (RTs) obtained in Blocks 3 (congruent) and 5 (incongruent) were submitted to repeated-measures ANOVA with type of aIAT (*false-memory aIAT* vs. *true-memory aIAT*) and congruency (congruent condition vs. incongruent condition) as within-subject factors. We note here that if participants can discriminate between true and false DRM memories the interaction between type of aIAT and congruency should be significant.

The main effect of congruency was significant: *F*(1,35) = 141.675, *p *< 0.001, η^2^ = 0.802. Overall, RTs were faster in the congruent than the incongruent condition (1173 vs. 1885 ms). The main effect for type of aIAT was not significant: *F*(1,35) = 0.538, *p *= 0.468, η^2^ = 0.015. Crucially for the hypothesis that we intended to test, we found a significant interaction between type of aIAT and congruency: *F*(1,35) = 4.948, *p *< 0.05, η^2^ = 0.124. The difference between incongruent and congruent conditions was greater in the *true-memory aIAT* than in the *false-memory aIAT* (788 vs. 637 ms). A closer inspection showed a marginal difference in RTs between the *true-memory aIAT* and *false-memory aIAT* tasks in the incongruent condition [1935 vs. 1834 ms, *t*(35) = 1.64, *p *= 0.055], whereas no significant effect was found in the congruent condition [1148 vs. 1197 ms; *t*(35) = −1.457, *p *= 0.077].

To test whether the significant interaction obtained in the within-subject ANOVA was affected by the presence of outlier observations or non-constant error variance (Fox, 1997) a linear mixed model was run with type of aIAT and congruency as independent variables and participants as random factor. Then we performed a case resembling bootstrapping analysis on model coefficients to derive a confidence interval for the interaction parameter. The 95% percentile confidence interval of the bootstrapped interaction parameter derived from 4000 repetitions ranged from 65.2 to 208.4 and did not include zero. Therefore we can conclude that there is a robust significant interaction between type of aIAT and congruency.

To test the accuracy of each aIAT in detecting true and false heard-related statements, for each participant we computed the *D* index for each aIAT (*true-memories aIAT* and *false-memories aIAT*). The mean *D* index was positive for both aIATs (*true-memories aIAT*, *D* score = 0.98 and *false-memories aIAT*, *D* score = 0.86) suggesting that both critical lures and targets might be efficiently discriminated from distracters. The one-tailed *t*-test, however, showed a greater *D* index for the *true-memories aIAT* than for *false-memories aIAT* [*t*(35) = 1.9218, *p *< 0.05, 95% CI = (0.0149, +∞)] and this result replicated the findings with raw RTs.

In sum, at the implicit level, both aIATs (*true-memories aIAT* and *false-memories aIAT*) showed an association both of the targets and critical lures with the logical dimension “true.” Faster responses were observed in the congruent condition, when sentences related to targets (*true-memories aIAT*) and critical lures (*false-memories aIAT*) were combined with the true factual statements than in the incongruent condition when they were combined with the false factual statements. This association was confirmed also by the positive value of the *D* index in both aIATs.

However, the significant interaction between type aIAT and congruency – re-marked by bootstrapping analysis (4000 repetitions) – and the greater *D* index in the *true-memories aIAT* than in the *false-memories aIAT*, indicated that a greater association was obtained when the logical dimension “true” was combined with the true memories (targets) than when they were compared with the false memories (critical lures).

## Discussion

The aim of the present study was to test whether false and true DRM memories can be discriminated at the implicit level using a novel instrument (aIAT; Sartori et al., 2008) developed to detect autobiographical memories encoded within the respondent’s mind/brain. The aIAT is a method based on indirect measures that permits us to detect which of two events is true for a given individual. In the aIAT, participants were requested to classify statements belonging to the true or false logical dimension and statements belonging to a true or false event by pressing one of two response keys. Faster responses are found when the statements related to the true event share the same motor response as the statements that are always true.

In our experiment, false memories were created with the DRM paradigm, originally developed by Deese (1959) and modified by Roediger and McDermott (1995). Several studies have demonstrated that the DRM paradigm produces high levels of false episodic memories which seem to convey the same recollective experience as true memories. Consistent with these results, we reported here that the false-alarm rates for the non-presented critical lures were not significantly different from the hit rate for the presented targets (0.84 vs. 0.90). The similar proportions of the true memories for presented words and the false memories for non-presented critical lures obtained in our experiment confirm that false and true DRM memories cannot be discriminated at an explicit level.

At an implicit level, the aIAT showed an association of both, presented targets and critical lures with the logical dimension “true.” At a first level of analysis, thus, the aIAT seems to be ineffective in detecting any difference between presented targets and critical lures. However, a closer inspection of our results showed that aIAT detected differences between presented targets and critical lures which cannot be distinguished at an explicit level. Indeed, we found a greater association of the logical dimension “true” with targets than with critical lures as indicated by the significant interaction obtained between type of aIAT and congruency and by the *D* index. Thus, the aIAT was effective to detect differences between true memories really encoded in the respondent’ mind and those incorrectly perceived as that (i.e., false memories). Specifically, our analysis shows that the aIAT is very effective in comparing true and false DRM memories although it is not suited to identify a single DRM memory as true or false “*per se*.”

The fact that both true and false-memories generated an implicit association with the dimension “true” can be potentially explained by taking into account that we have a greater tendency to use “remember” judgments for presented targets than for non-presented critical lures in a DRM task (Tulving, 1985; Roediger and McDermott, 1995). “Remember” judgments are made when the participant can recall specific details about an event’s prior occurrence. “Know” judgments are made when the participant cannot recall specific details about the event’s prior occurrence, but she/he nevertheless believes that it has occurred (e.g., it is familiar, but there is no specific remembrance of its prior occurrence). Gallo (2006), for example, in an analysis of 32 experiments which used Remember/Know judgments (R/K judgments) in the DRM paradigm, found significantly more “Remember” judgments for true memories (mean = 0.47) than for critical lures (mean = 0.40). In addition, whereas “Remember” judgments given to related lures were greater than those given to unrelated lures (mean = 0.06), “Know” judgments of true and falsely recognized words did not differ, or were greater for related lures. These results suggest that false memories for critical lures might have a phenomenological status similar to that of true memories but actually located between true memories and false memories for non-critical lures. This may explain the differences in absolute values of the IAT effect and the *D* index found in our experiment. In other words, participants assigned explicitly a high belief value to the false memories. Thus at the explicit level they reported their subjective high belief. This was not different for true and false memories, and thus did not provide information to help distinguish between true and false DRM memories. Conversely, the distinction between true and false memories was clear when the aIAT was used, which implicitly measures the association actually present in the participants’ memory, rather than their subjective beliefs.

It is also possible to interpret our findings in the framework of the fuzzy trace theory (Reyna and Brainerd, 1995). According to this theory there are two types of memory representation: gist and verbatim. A gist representation is fuzzier (hence the name of the theory) and includes the semantic content of an event. On the contrary, a verbatim representation is more precise and includes the physical structure of the event. False memories rely mainly on the gist (Brainerd and Reyna, 2005). This theoretical proposal would suggest that the difference detected with the aIAT might depend on the differences in type of representation accessed for true and false memories. They further suggest that explicit tests might not be sensitive enough to reveal this difference, whereas implicit measures might represent a more appropriate test.

It must be emphasized that the specific goal of the present study was to validate the aIAT as an instrument to detect differences between true and false DRM memories at the group level. Indeed, we detected presented targets from critical lures by comparing the averages of the *D* index obtained in two separate aIATs. This analysis is statistically sound only when applied to the *D* indexes measured from a set of subjects. For this reason, no definitive conclusion can be drawn on whether present experimental results indicate possible significant differences also at the individual level. One possible manner to extend our procedure to the individual level could be to directly compare true and false DRM memories in a single aIAT. Further studies are needed to validate the feasibility of this proposal.

In summary, in the present paper we reported differences between true memories for presented targets and false memories for critical lures in a DRM task using the aIAT. These results are relevant because they demonstrate that although true and false DRM memories are indistinguishable at the explicit level they can potentially be detected at the implicit level.

## Conflict of Interest Statement

The authors declare that the research was conducted in the absence of any commercial or financial relationships that could be construed as a potential conflict of interest.
